# Large Area Patterning of Nanoparticles and Nanostructures: Current Status and Future Prospects

**DOI:** 10.1021/acsnano.0c09999

**Published:** 2021-04-08

**Authors:** Hannah-Noa Barad, Hyunah Kwon, Mariana Alarcón-Correa, Peer Fischer

**Affiliations:** † Max Planck Institute for Intelligent Systems, Heisenbergstrasse 3, 70569 Stuttgart, Germany; ‡ Institute of Physical Chemistry, University of Stuttgart, Pfaffenwaldring 55, 70569 Stuttgart, Germany

**Keywords:** nanoparticle patterns, parallel methods, large-area patterning, template nanopatterns, nontemplate nanopatterns, nonlithographic methods, chemical/physical patterning, organic templates, magnetic/electric field patterning

## Abstract

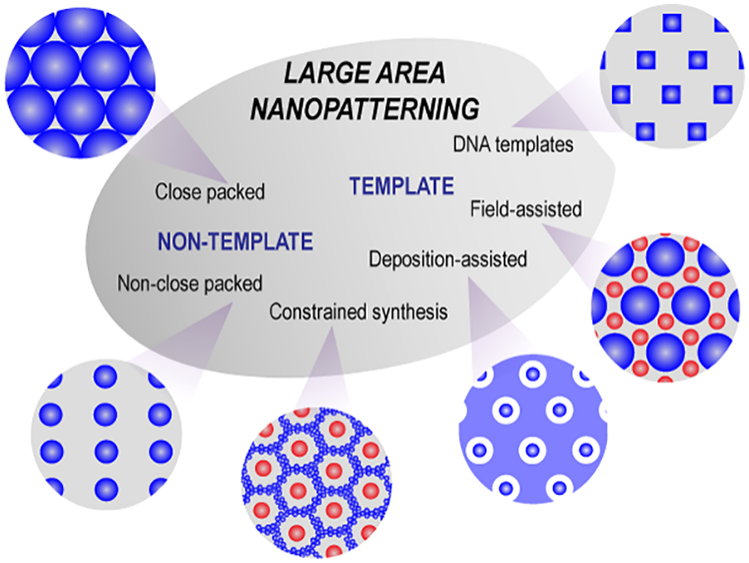

Nanoparticles possess exceptional optical, magnetic, electrical, and chemical properties. Several applications, ranging from surfaces for optical displays and electronic devices, to energy conversion, require large-area patterns of nanoparticles. Often, it is crucial to maintain a defined arrangement and spacing between nanoparticles to obtain a consistent and uniform surface response. In the majority of the established patterning methods, the pattern is written and formed, which is slow and not scalable. Some parallel techniques, forming all points of the pattern simultaneously, have therefore emerged. These methods can be used to quickly assemble nanoparticles and nanostructures on large-area substrates into well-ordered patterns. Here, we review these parallel methods, the materials that have been processed by them, and the types of particles that can be used with each method. We also emphasize the maximal substrate areas that each method can pattern and the distances between particles. Finally, we point out the advantages and disadvantages of each method, as well as the challenges that still need to be addressed to enable facile, on-demand large-area nanopatterning.

The two-dimensional (2D) assembly of nanoparticles (NPs) on surfaces has led to discoveries and progress in fields such as plasmonics, biological sensors, electronics, and optics.^1−6^ These NP arrays are either directly used for a specific application or can be modified to obtain more complex ordered structures, as shown in Figure 1a–c.^7,8^ The assembly of NPs with high precision and uniformity is very important for applications where the periodicity affects functionality.^9,10^ As such, accuracy and reproducibility have become crucial parameters when patterning NPs on surfaces. Controlling the spacing between NPs or ensuring that arrays of nanostructures are all aligned on a surface is, however, far from trivial.

**Figure 1:**
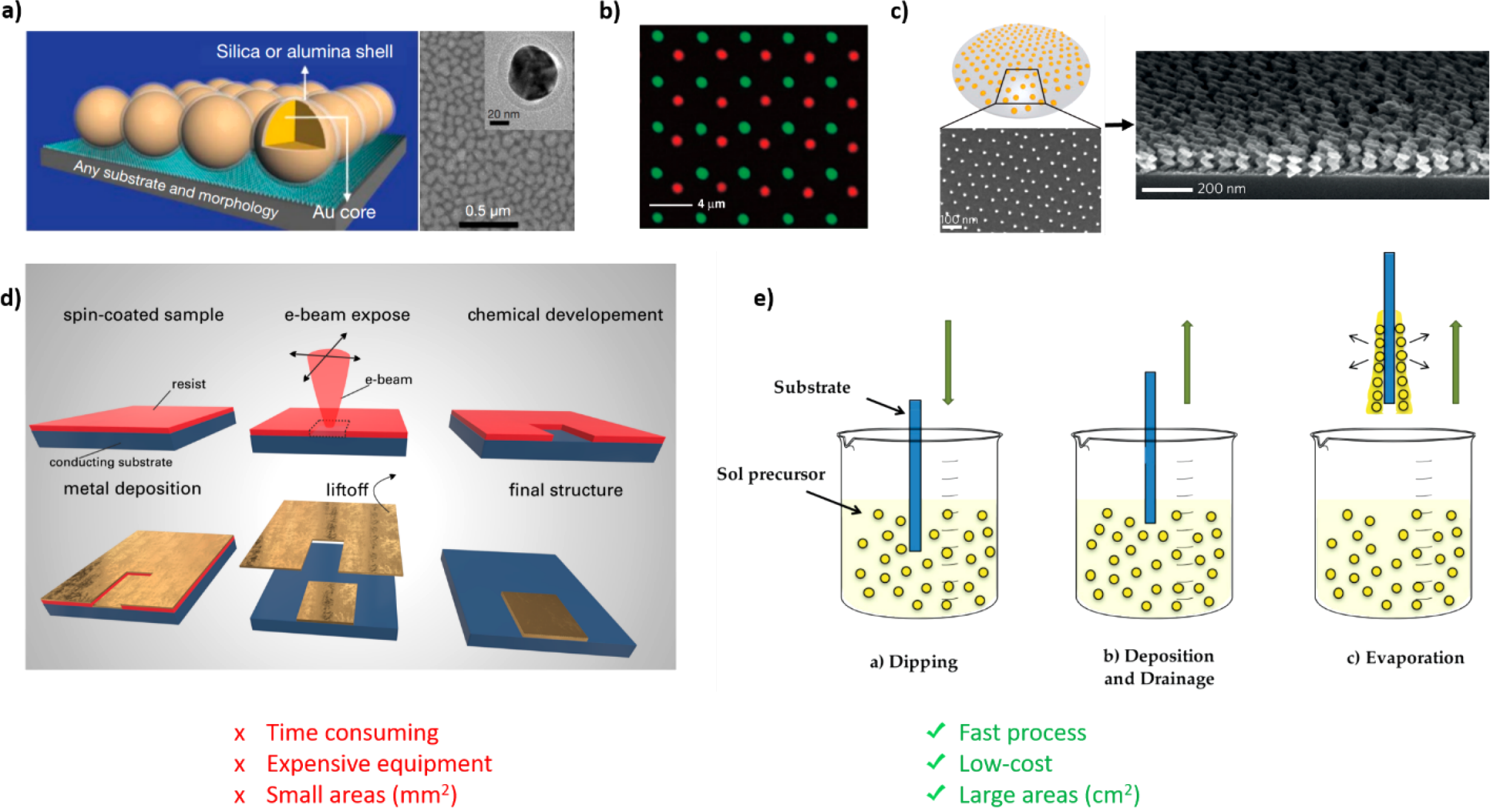
Examples of different applications where large-area nanopatterns are utilized. (a) SiO_2_-coated Au NP arrays for surface-enhanced Raman spectroscopy. Reproduced with permission from ref (5). Copyright 2010 Springer Nature. (b) Au NP arrays used to direct the assembly of particles modified by oligonucleotides, here, with red and green fluorophores coupled to the surface by oligonucleotides with specific binding sequences. Reproduced with permission from ref (6). Copyright 2002 The American Association for the Advancement of Science. (c) Au nanopatterns on 2 in. Si wafers used as seeding layers for growth of helical nanostructures by glancing angle deposition (GLAD). Reproduced with permission from ref (8). Copyright 2013 Springer Nature. The fabrication steps involved in (d) sequential patterning with e-beam lithography, compared with (e) a much simpler parallel dip-coating process. Reprinted (adapted or reprinted in part) with permission under a Creative Commons Attribution 3.0 License from ref (17). Copyright 2013 American Chemical Society.

Commonly used nanopatterning approaches include electron beam lithography and ion beam lithography (Figure 1d).^11,12^ While these methods allow for extraordinary precision and reproducibility and possess high resolution (down to 10 nm), they also have a number of drawbacks. First, the substrate must be pretreated and post-treated during the patterning process, adding a resist layer and later removing it with chemical etchants. Second, the procedures are extremely time-consuming, as the electron or ion beam has to be rastered across the surface, and the pattern is sequentially written point-by-point. As scanning the electron beam at high resolution is a slow process, this leads to the third main disadvantage, the limitation of the patterning to a small area on a substrate (typically only up to few square millimeters). Furthermore, the equipment is relatively expensive to purchase and to operate.

In order to pattern larger surface areas and with higher throughput, one must turn to parallel methods. These are often also technically much simpler and therefore do not require sophisticated setups or expensive equipment. Many parallel techniques have utilized the wetting and self-assembly properties of NPs, sometimes in conjunction with templated substrates, to form large-area NP arrays on areas as large as tens of square centimeters.^13^ Their advantage is that the patterns can be obtained quickly with simple laboratory equipment and on many different substrates (Figure 1e).^14^ However, the precision in NP assembly and the reproducibility of the pattern are more challenging to control with parallel methods.

In this review, we focus on parallel nanopatterning methods that can form close-packed as well as non-close-packed patterns on large-area substrates. We also discuss the procedures that are used to control the spacing between the NPs in large-area patterning. It is shown that the use of templated (prestructured) substrates significantly increases the control over the interparticle distance. We try to focus on particles that are in the nanometer range; however, we also mention if larger particles can be used. Furthermore, we attempt to give an overview of the materials (metals, semiconductors, insulators, *etc.*) and substrates (glass, Si wafers, *etc.*) that have been used, as well as the sizes of the covered areas. We provide an assessment of the general advantages and drawbacks of a number of parallel nanopatterning techniques (Figure 2).

**Figure 2:**
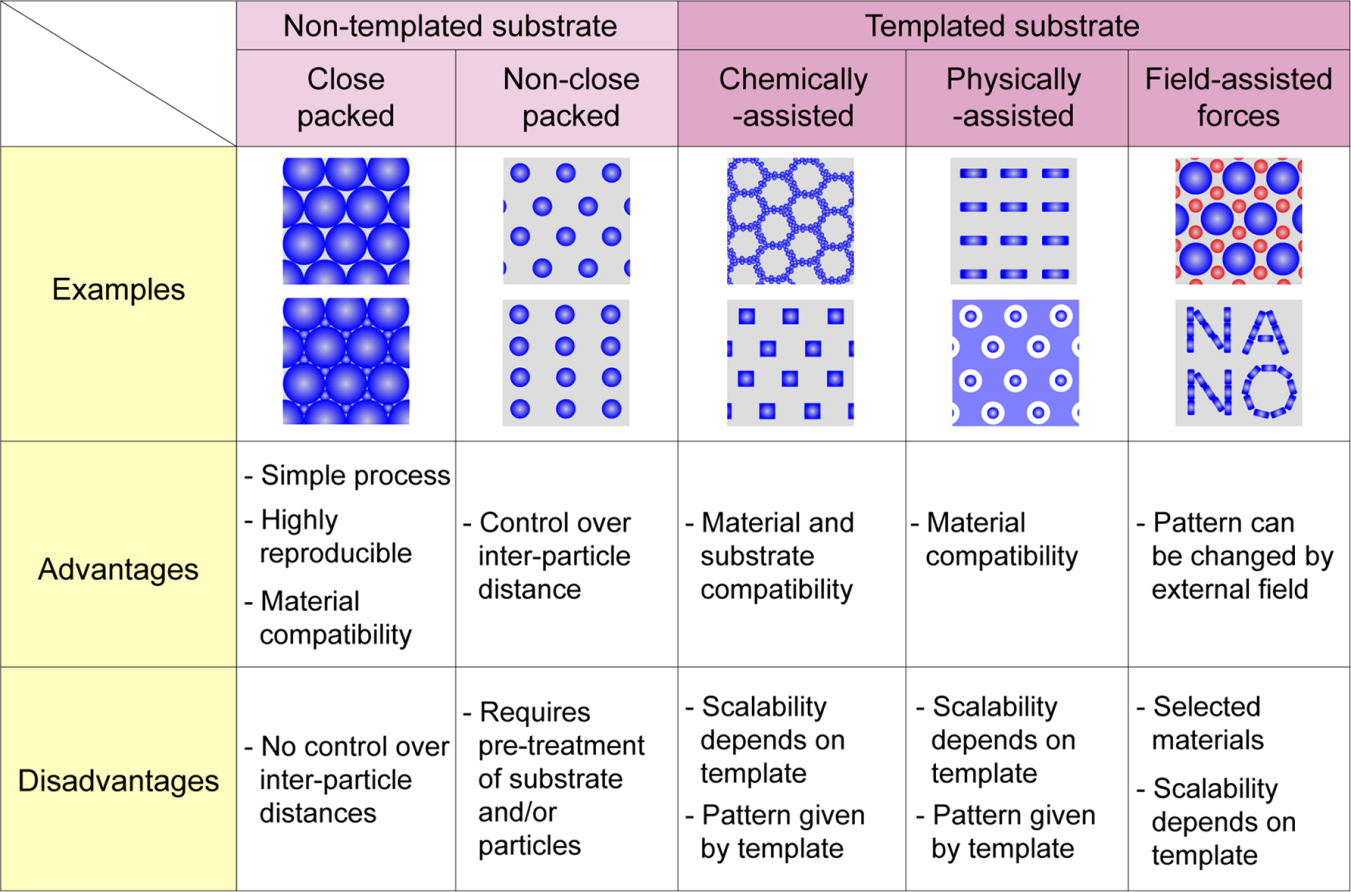
Summary of the advantages and disadvantages of the different types of methods used for the parallel formation of large-area nanopatterns. We emphasize the differences between templated and nontemplated substrates. Templating requires a preprocessing step to modify the substrate before the nanopatterns form.

## Nontemplate-Assisted Patterning

**Figure 3:**
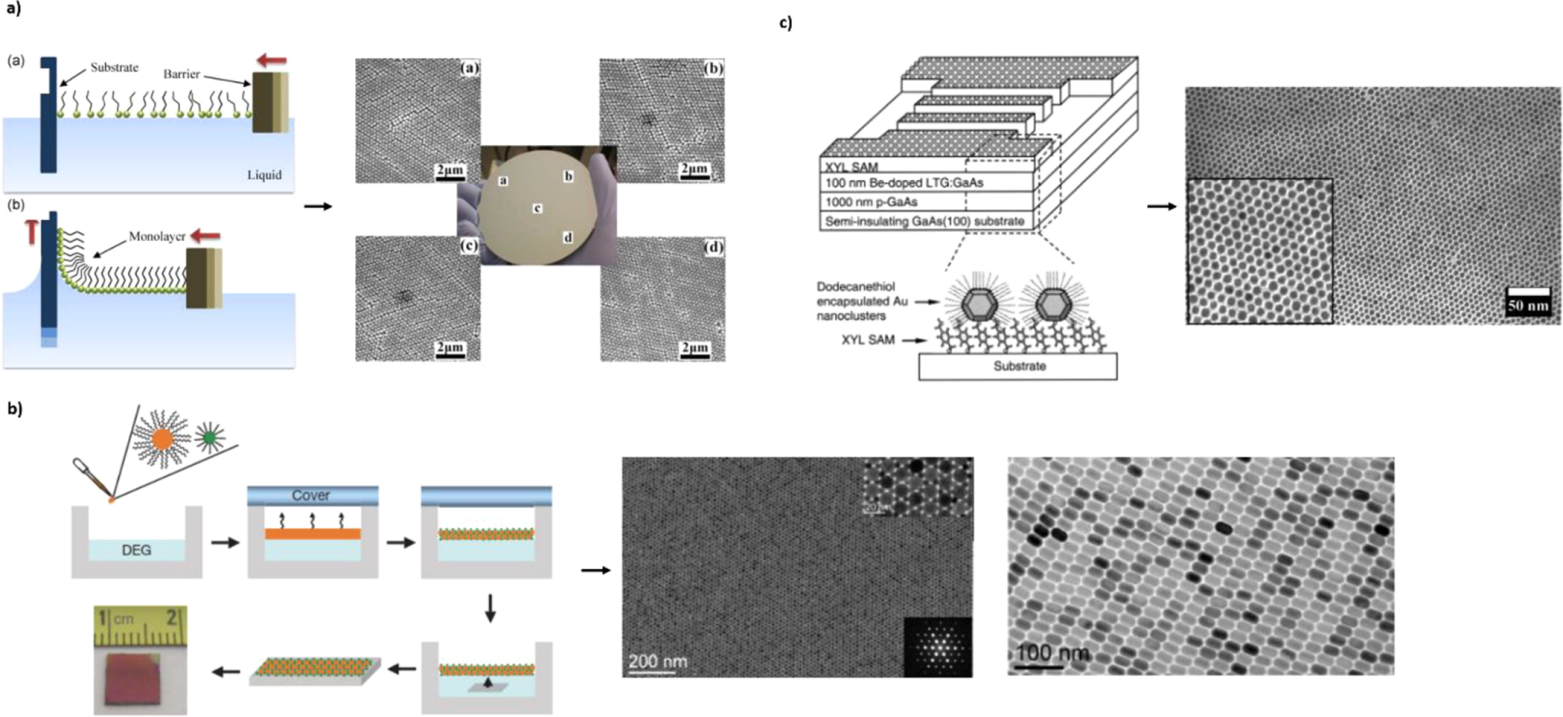
Nontemplated close-packed patterns. (a) Langmuir–Blodgett method^35^ utilizes the liquid/air interface where a layer of particles forms and is then compacted and deposited onto a substrate, for example, a full Si wafer with patterns of silica particles. Reproduced with permission from refs (35) and ref (32). Copyright 2014 Springer Nature and copyright 2008 AIP Publishing, respectively. (b) Liquid/air method, where a NP pattern forms at the liquid/air interface and a substrate (placed under the layer prior to its formation) is then transferred together with the layer on top of it. The liquid then evaporates leaving behind, in this case, a superlattice of metal oxide spheres and rods. Reproduced with permission from refs (16) and ref (17). Copyright 2010 Springer Nature and Copyright 2013 American Chemical Society, respectively. (c) Assembly of Au NPs on a thiol-modified GaAs surface for electronic semiconducting properties. Reproduced with permission from ref (34). Copyright 2000 AIP Publishing.

Two types of patterns can be fabricated on bare (nontemplated) solid substrates: close-packed and either ordered or disordered non-close-packed structures. In a close-packed structure, the particles form a dense array, touching each other. The pattern, typically, is a highly ordered monodisperse 2D hexagonal close-packed assembly, where the particles have a minimum interparticle distance (see Figure 3). For the non-close-packed structure, the NPs in the pattern are separated by varying distances and can assume many types of 2D surface patterns, including hexagonal, cubic, rectangular, and additional symmetries.^15^

### Close-Packed Patterns

Close-packed monolayers can, for instance, be formed by evaporation at the liquid/liquid or liquid/liquid/air interfaces.^16,17^ In these methods, a high vapor pressure liquid (*e.g.*, hexane or toluene), containing the NPs, is poured over a low vapor pressure liquid with a higher density (*e.g.*, diethylene glycol). The particles self-assemble at the liquid/liquid interface, while the higher vapor pressure liquid evaporates, forming a pattern suspended at the liquid/air phase. The self-assembled particle film forms due to the interaction between the different nanocrystal facets of the NPs in the original suspension. A substrate placed beneath the particle suspension is then gently lifted up and out, usually at an angle, and the pattern is transferred to the substrate surface. If needed, a second lift-off can transfer the monolayer from the substrate to be deposited elsewhere. Many different particles and substrates have been used in this method to form nanopatterns, as indicated in Table 1 (and its references).

**Table 1:** Summary of the Prevalently Used Nontemplated Patterning Methods

methods	types of substrates	types of particles (materials, shape, size)	interparticle distance	reported patterned areas	refs
liquid/liquid and liquid/air interfaces	glass, Si, TEM grids, semiconductors (GaAs)	polymers, metals, oxides, spheres, rods, and platelets, with sizes of 5 nm to hundreds of microns	usually close-packed but can control distance with substrate insertion/removal angle	up to 2 cm^2^	(16,17,33)
dip-coating	glass, Si	metal spheres and semiconductor (QD) spheres, several nm to microns	close-packed	up to 2 in. wafers	(18−20)
Langmuir–Blodgett	glass, Si	SiO_2_, polymer spheres, hundreds of nanometers to hundreds of microns	usually close-packed (distances can be controlled by ligands or polymer shells)	up to 3 in. wafers	(15,25,42)
BCML	Si wafers, glass, ITO	mainly Au (Pt, Ni are possible but difficult), spherical NPs, 5 to 15 nm	from 30 to 300 nm	up to 4 in. wafers	(38−40)
spin-coating	ITO	mainly Au spheres and rods, but also CdSe NPs, 10–70 nm	close-packed up to 60 nm	several square centimeters	(41)

Alternately, dip-coating may be used, where a substrate is immersed in a solution containing particles and then continuously extracted at a controlled speed to yield a close-packed monolayer of particles that are compacted by the fluid meniscus surface tension.^18−20^ In this case, the particle film thickness and formation depend on several parameters, namely, the liquid viscosity, surface tension, and the drag force (resulting from the substrate withdrawal).^21,22^ In order to form thinner particle films, the withdrawal speed needs to be slowed down so that the drag force is reduced to a minimum, lifting out less liquid onto the substrate, which is valid for >1 μm particles. However, when the withdrawal rate is equal to the particle array formation rate, then arrays can be formed independent of particle size.^23^ Furthermore, highly volatile liquids can evaporate very quickly from the surface of the substrate while it is being extracted, which may lead to a gradient in the surface tension of the particle film. This may ultimately lead to the collapse of the particle film. Hence, a gentle balance between the withdrawal rate and the liquid’s vapor pressure must be considered. The substrate size used for dip-coating is essentially unlimited (the technique is frequently used by the fabric industry to dye fibers)^24^ and can change based on bath size, desired particle film (homogeneous, gradients, or patterned coverage), and processing liquids.

The Langmuir–Blodgett technique, in particular, has been used to form monolayers of particles on surfaces (Figure 3a).^25^ In this case, both surface tension and evaporation from the liquid/air interface are utilized for the nanopattern formation. In the Langmuir–Blodgett method, amphiphilic molecules or functionalized particles are dissolved in a volatile liquid and then spread over a pure deionized water phase.^26^ The water pH and temperature must be controlled to induce formation of a defect-free film. The solution containing the particles forms a monolayer on the surface of the water. The surface pressure of the monolayer is then increased by an external barrier placed in the water to form a close-packed film, whereas, in the meantime, the volatile solvent evaporates. The film is then transferred onto a substrate by raising it slowly out of the water bath. The substrate is generally placed normal to the film, and as the removal is similar to dip-coating, the elements governing the film formation, such as liquid viscosity, are also determining factors for the success of the deposition. Binary colloidal monolayers with a close-packed monolayer of larger spheres with a surrounding superlattice of smaller particles can also be prepared with the Langmuir–Blodgett method. By adjusting the size ratio between large and small particles and optimizing experimental conditions, various stoichiometries at the interface can be controllably fabricated.^27−29^ We will now give an overview of materials and substrates that have been used to form close-packed large-area nanopatterns, fabricated by the aforementioned methods.

Many different types of materials can be synthesized as NPs;^30^ however, assembling them into patterns is a more complex task. Nevertheless, close-packed monolayers as well as superlattices of various combinations of nanospheres and nanorods including, Au, Pd, Fe_2_O_3_, and NaYF_4_, have first been formed at the liquid/liquid/air interface^17^ and subsequently have been transferred onto substrates (Figure 3c). In this case, the patterns formed irrespective of material, but the concentrations of the NPs and their stabilizing ligands had a major influence on the particle assembly.^17^ Using Langmuir–Blodgett deposition, oxides, such as SiO_2_ of varying particle sizes have been patterned as close-packed layers.^31,32^ Semiconductor NPs (quantum dots) have been used to form 2D close-packed monolayers, for example, PbSe 2D superstructures formed at the liquid/liquid interface^18^ and CdSe monolayers by dip-coating.^19,20^ Close-packed LnF_3_ and LiYF_4_ nanoplatelet layers have been formed by interfacial assembly.^33^

Many substrates are used for close-packed patterning; however, several applications demand specific substrates. GaAs was used as an activated substrate on top of which a monolayer of Au cluster arrays was prepared by dip-coating the substrate in a monolayer of the Au particles that formed at the liquid/liquid/air interface (Figure 3b).^34^ Close-packed nanopatterns have also been formed directly by dip-coating conducting Sn-doped In_2_O_3_ (ITO), which allowed for measurements of their surface photovoltage.^19,20^ Transferring the close-packed layer of NPs from a liquid interface permits the use of applications involving unusual substrates, like carbon-coated Cu transmission electron microscopy (TEM) sample grids.^17,18^

### Non-close-Packed Patterns

The simplest non-close-packed patterns are those that form without any particular order, for example, by random sequential adsorption (RSA).^36^ By immersing a surface-modified substrate in a NP solution and tuning the ionic strength, it has been possible to obtain Au nanopatterns with varying interparticle distances; however, no long-range order was seen in this case.^37^

**Figure 4:**
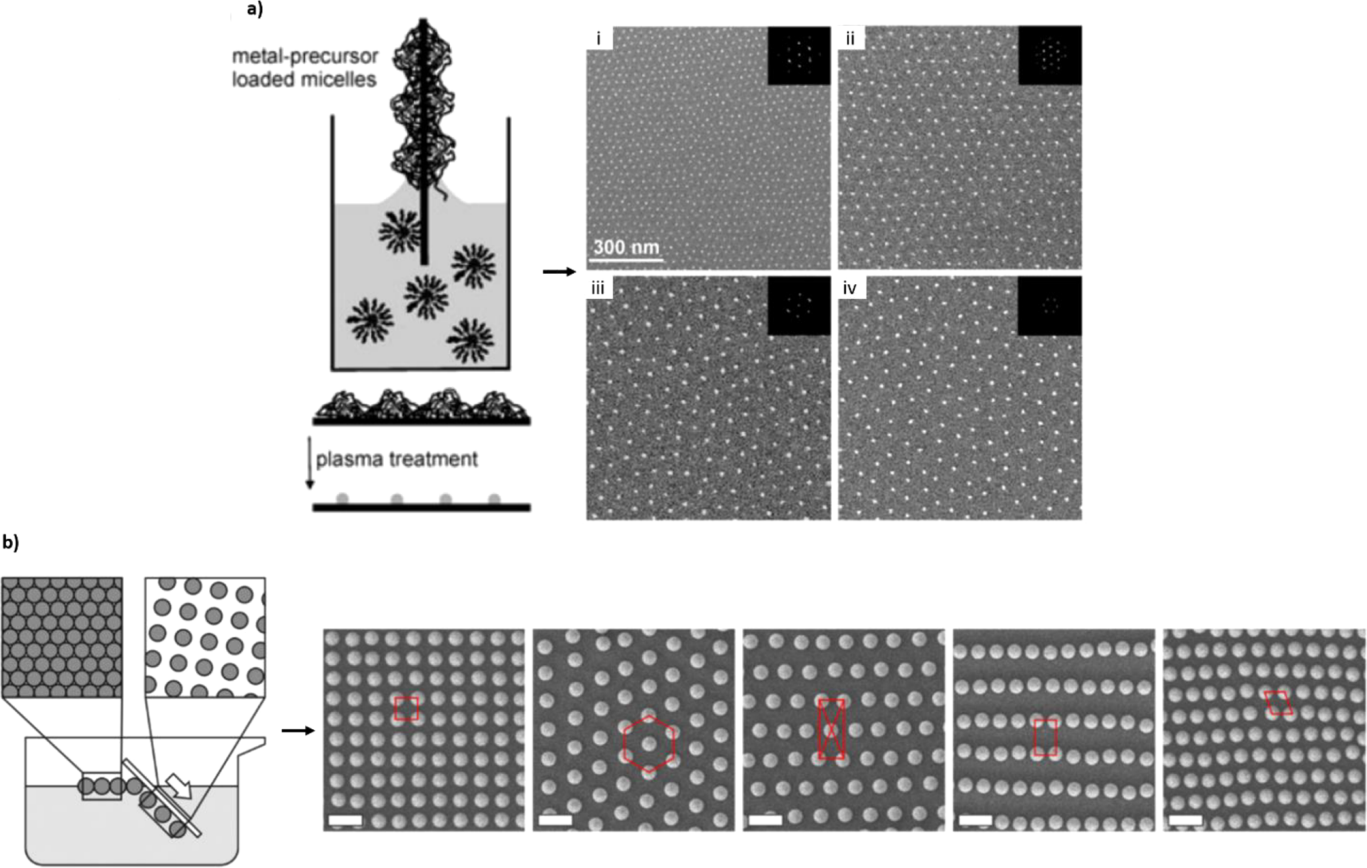
Nontemplated non-close-packed patterns. (a) Block copolymer micelle nanolithography (BCML), where block copolymers of different lengths are impregnated with metal salts and then deposited by dip-coating (or spin-coating) onto a substrate. The polymer is subsequently removed by plasma treatment, which also reduces the corresponding salts to metallic NPs. The distances between the NPs are determined by the length of the block copolymer. Here, in images i–iv, the variations in distance are given by variations in polymer chain lengths of the diblock copolymers polystyrene(*x*)-*block*-poly(2-vinylpyridine)(*y*), where the *x* and *y* values are 1350 and 400, respectively, for image iv. Reproduced with permission from ref (38). Copyright 2003 IOP Publishing. (b) Utilization of the water/air interface and specific angles and stretching factors (of substrate insertion or removal) to form different Bravais-like nanopatterns, including squares, hexagons, rectangles, and more. The scale bars are 1 μm. Reproduced from ref (15). Copyright 2019 American Chemical Society.

In contrast, controlling the interparticle distance is much more challenging. Of prominence in this regard is patterning using block copolymer micelle lithography (BCML).^38−40^ Inverse micelles are formed in a block copolymer solution by self-assembly in a suitable solvent. Addition of metal salts localizes the metal ions inside the hydrophilic core of the micelle. The solution containing the micelles can then be spin- or dip-coated onto a substrate, where the micelles form a close-packed monolayer on the substrate. The spin-coating is followed by a plasma treatment to reduce the salt to a metallic NP, while at the same time burning away the organic polymer shell. The chain lengths of the block copolymers control the size of the micelle and hence the center-to-center distance between the micelles. Larger chains will lead to larger interparticle distances between the NPs after plasma treatment (Figure 4a). The salt concentration determines the size of the resulting NPs. In a different approach,^41^ spherical Au NPs and nanorods were synthesized with conventional methods, and then molecular ligands were exchanged for polymer ligands to form shells around the particles. Subsequently, the ligand-modified particles were spin-coated on large-area substrates, and the polymer shell was burned off with a plasma etch. The spherical NPs formed ordered patterned surfaces, where the particle-to-particle distance depended on the length of the polymer ligand shell surrounding them.

Nanopatterns can be formed by removing the substrate from the particle suspension at a specific angle, such that the particle layer that has been formed at the liquid/air interface transfers onto the substrate (Figure 4b).^15^ This allows for the formation of regular 2D lattices. The interparticle distances can be tuned by the substrate removal angle and, as such, may form lattices that have enough room between the particles for other particles to be deposited interstitially. To achieve this, the substrate with the 2D lattice is again inserted into a particle suspension; however, this time the suspended particles are smaller, suitable to fit between the patterned particles that are already deposited on the substrate. The larger particles remain on the surface due to van der Waals interactions with the substrate.^42^ The interstitial particle sizes are limited because of steric hindrance. However, the number of particles that can occupy interstitial spaces is a function of the Langmuir force applied to the liquid interface during the process. Alternately, one can also synthesize metal or oxide NPs that are coated with a polymer shell and deposit these core–shell particles *via* the Langmuir–Blodgett method.^42,43^ In all cases of the core–shell, polymer-coated NPs, or the block copolymer micelles, plasma treatment must be used to remove the polymer layer and to leave “bare” NPs on the substrate as the nanopattern unless a polymer pattern is required. A major issue that may arise during the formation of large-area patterns at the liquid/air interface is the deformation of the pattern while the solution evaporates. The accompanying attractive capillary forces can cause the collapse of the pattern. Increasing the adhesion of the particles to the substrate by surface treatment or by increasing the contact area between the particles and the substrate can prevent the collapse of the pattern.^15^

The BCML method has been used in conjunction with metallic NPs, mainly Au, but also Ag, Pd, Pt, and Ni.^40,44^ However, the shape of the NP seems more restrictive, as neither method has produced nanopatterns containing nonspherical NPs. The nanopatterns have been formed on Si, SiO_2_, quartz, glass, and ITO substrates (Table 1), where the size of the substrate does not present a real limitation. Practically, the distance of NPs in large-area patterns is not limited by the type of substrate used but depends on the material (Au or other metals). Further methods must be developed to increase the diversity of materials as well as NP shapes to form non-close-packed patterns.

The nontemplated methods have many advantages as they can be performed with simple laboratory equipment and do not require preceding steps to form patterns on large areas; however, it is difficult to control the distances between the NPs or the produced patterns, which can be achieved with help of a template, as discussed next.

## Template-Assisted Patterning

Templates can be made of very different kinds of materials and are usually produced using slow, laborious nanofabrication methods such as e-beam lithography or external fields. However, once a template is prepared, it can be used multiple times and assist in the formation of precise and diverse types of nanopatterns *via* a parallel patterning method. Nanoparticle patterns can be formed on the template by electrostatic forces, controlled dewetting, confined deposition and growth, or selective etching techniques, which will be discussed in this section. In addition, external fields such as magnetic, electric, and even light can assist the patterning process to provide precise positioning of NPs in desired locations on the template. In this section, we do not discuss the fabrication of the template structures themselves but instead focus on template-assisted nanoarrays using parallel patterning methods.

### Patterns Assisted by Chemical and Physical Interactions

Some of the approaches for preparing particle nanopatterns are based on the use of molecules that are chemically coupled or grafted to a surface. These grafted molecules work as a bridge to capture the NPs in an organized way and give rise to a pattern of NPs on the surface. In some patterning approaches, the surface first needs to be treated such that molecular structures (patches) can be deposited on the surface to facilitate the patterning with NPs. These patches can be formed either by self-assembly or using a template prepared by a serial (slow) method. In this section, we classified these methods into fully parallel methods, where the system organizes itself using environmental conditions and self-assembly of the components, and later describe those methods that need some templating before the parallel formation of the particle pattern. We do not discuss serial methods that are used for templating substrates such as e-beam lithography, as these methods have been extensively discussed in the past.^12,45^ Finally, we describe a method that uses patterned substrates for the fabrication of more complex patterned nanostructures.

#### Fully Parallel Methods

Organic molecules such as polymers, peptides, and DNA, among others,^46^ are useful in soft lithographic methods for the patterning process. Self-assembled molecular crystalline structures can also guide the *in situ* synthesis of NPs. For instance, genetically engineered hollow proteins (chaperonins) can self-assemble into a 2D structure after crystallization on a solid surface to form a template as large as 20 μm in diameter and can thus be used for the binding or constrained chemical synthesis of NPs. Chaperonins engineered with thiol or His-tag molecules are able to scavenge small Au NPs or quantum dots into thiol-modified binding motifs.^47^ Nanoparticles can thereby be positioned at 16 nm interlattice distance (center-to-center) with ±2 nm accuracy, determined by the protein crystal lattice. When the chaperonins are instead modified with a His-tag, they enable the *in situ* synthesis of Pd–Ni and Pd–Co NPs.^48^ However, the synthesized NPs, and therefore the patterned features, are found to be highly polydisperse.

DNA has also emerged as a promising platform to realize organized NP patterns. The specificity of a base pairing technique, exemplified in DNA origami,^49,50^ has enabled the supramolecular assembly of gold NP chains^51,52^ and quantum dots^53,54^ on areas ranging from hundreds of nanometers to hundreds of microns. Even nanoarrays with a single quantum dot at each designed position can be generated by one lithography step combined with DNA origami.^53^ Such molecularly precise templates show great promise for the positioning of NPs from a solution onto solid surfaces. Furthermore, they have the advantage of specificity, when compared to lithographic stamps, where structural order is not as easily achieved at the nanoscale. When combined with polymer-based templates, DNA technology can be most effectively used for large-area patterns, as discussed in the next subsection.

Similar to DNA origami, the use of amphiphilic molecules permits the formation of nanoscopic lattices without the need for the serial writing of templates. Amphiphilic molecules at the water/air interface form stripe- and channel-like nanolattices caused by wetting instabilities using the Langmuir–Blodgett technique. During the process, the nanolattices are transferred onto a mica substrate. This method has been used to create hydrophilic channels of ∼200 nm in width with a ∼800 nm hydrophobic barrier by a rapid extraction of the substrate and a low monolayer surface pressure. By choosing a suitable combination of nanoparticle and amphiphile, a linear array of NPs can be formed, as was demonstrated using Au_55_ clusters or dye molecules.^55^

Block copolymers can form periodic patterns with feature sizes ranging from 10 to 50 nm as they are able to self-assemble into supramolecular architectures with an accuracy and an efficiency higher in an e-beam lithographic process.^56^ Studies on the kinetics and thermodynamic assembly of defect-free patterns have demonstrated pathways to make nearly defect-free patterns over hundreds of square centimeters.^57,58^ For example, advances in nanophotonics^59^ and superhydrophobic surfaces^60^ have emerged from the self-assembly of block copolymers on substrates.

**Figure 5:**
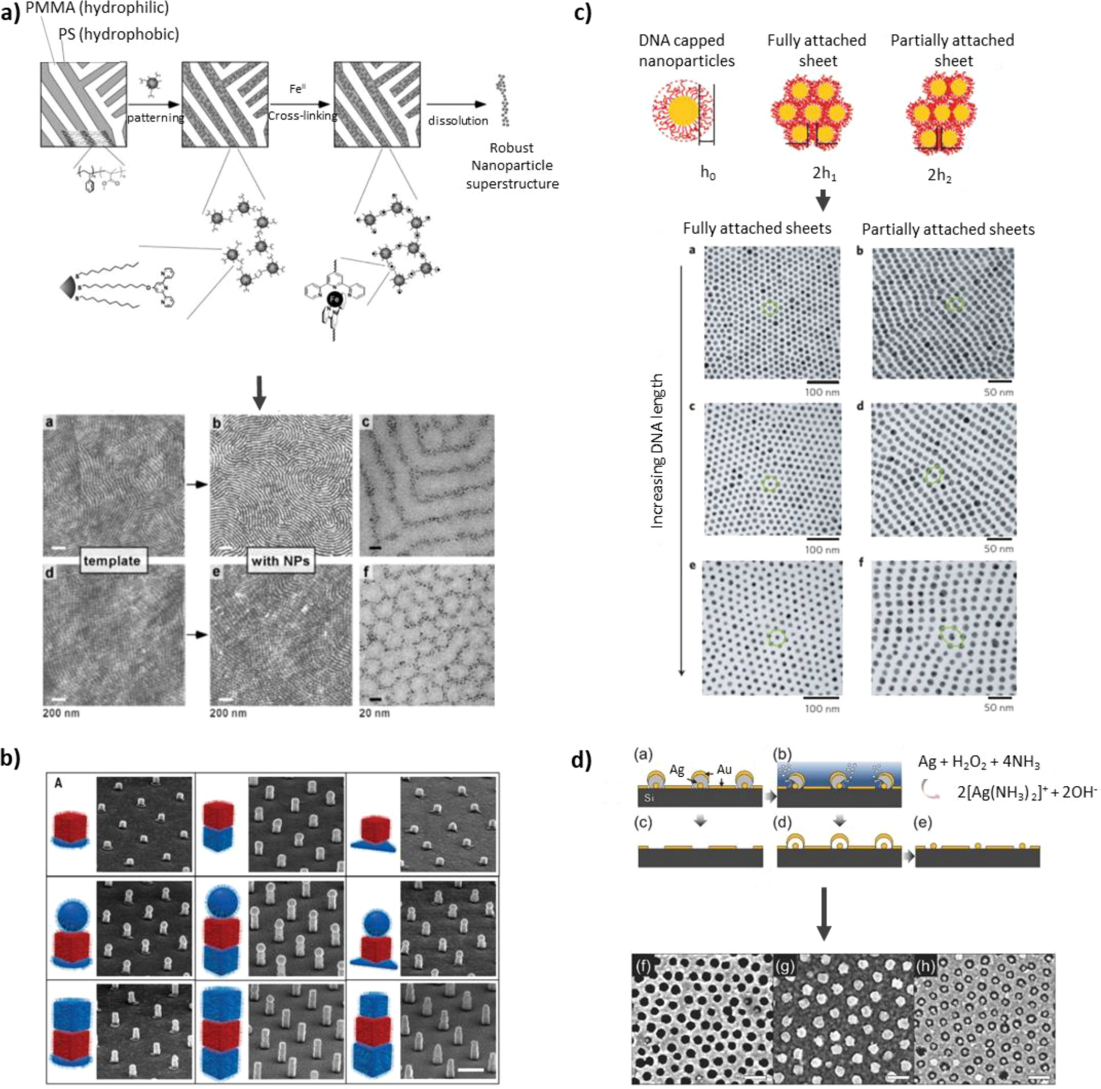
Template assisted patterning. (a) Block copolymer template patterning using coordination chemistry for cross-linking and removal of the polymer. AFM (a,b,d,e) and electron microscopy images (c,f) of line or dotted patterns. Reproduced with permission from ref (61). Copyright 2005 John Wiley and Sons. (b) Two or three NP layer superlattices, created by compatible DNA single strand functionalization on the individual NPs. Scale bar 300 nm. Reproduced with permission from ref (62). Copyright 2018 The American Association for the Advancement of Science. (c) DNA-capped NPs and their deformation at different states of the lattice formation (colloidal state, fully, or partially attached sheet of NPs). TEM images of the sheets formed using 15 (a,b), 30 (c,d), or 90 (e,f) DNA bases as modification for the NPs. Reproduced with permission from ref (63). Copyright 2009 Springer Nature. (d) Multifunctional nanopattern. Fabrication scheme (a–e) and scanning electron microscopy images of resulting nanoholes (f), nanodomes (g), and nanorings (h). Scale bar 100 nm. Reprinted (adapted or reprinted in part) with permission under a Creative Commons CC BY License from ref (84). Copyright 2015 John Wiley and Sons.

Nanoparticles have also been patterned without requiring the need to fabricate a stamp when block copolymers are used to obtain complex one- or two-dimensional arrangements of NPs (Figure 5a). As an example, polystyrene-*block*-poly(methyl methacrylate) (PS-*b*-PMMA) self-assembly can be used for the fabrication of a NP pattern when a thin film of a PS-*b*-PMMA diblock copolymer self-organizes as PMMA cylinders in a PS matrix. The block copolymer is placed on a hydrophobic surface, and after annealing, the film self-organizes into PMMA cylinders parallel to the surface. By tuning the annealing temperature and the substrate hydrophobicity, the casted films will form either lines, hexagons, or dot-like patterns. Once the substrate is patterned with the block copolymer, terpyridine-functionalized gold NPs can be fixed onto the PS site by complexing the terpyridine ligands to the polymer with metal salts. The polymer can finally be dissolved to leave only the NP pattern on the surface.^61^

#### Combined Serial and Parallel Patterning

Instead of proteins, one can also use polymer layer substrates that have been patterned using a slow lithographic method. As an example, Au surfaces can be selectively exposed by a hole-patterned PMMA (resist) layer affixed to a Au-coated substrate by e-beam lithography, exposing the Au surface on specifically designed areas (pores). Each pore is then decorated with DNA single strands that can trap colloidal NPs, which are functionalized with complementary DNA strands. This process can be repeated with multiple particles, each decorated with adequate single strands, to form a 3D pattern in a layer-by-layer manner, before removing the PMMA template and exposing highly organized multiparticle arrays (Figure 5b).^62^ Similarly, with the help of DNA chains functionalized onto Au NPs, sheets of freestanding superlattices have been patterned. The interparticle spacing is highly tunable and controlled by modifying the length of the DNA chains (Figure 5c).^63^

Nanoimprint lithography (NIL) is widely used for large-area patterning.^64,65^ Although it requires serial e-beam lithography in a first step to fabricate a master mold, it can then be repeatedly used to replicate the mold pattern. The imprint resist is coated on the substrate, and after the master mold has been mechanically pressed into the resist, it is cured by heat or UV light. Resolution to sub-20 nm has been achieved. Conventional NIL usually requires subsequent steps such as deposition and etching. However, recently, a UV-curable resin that includes TiO_2_ NPs was used to directly pattern materials onto the substrate, showing that NIL has the potential to become a single-step patterning method once a master mold has been made.^66,67^

Capillary assembly, which is usually combined with a doctor-blade approach, can also be applied for the patterning of NPs.^68−71^ Here, a template that works as a particle trap is necessary to utilize capillary forces. A solution containing NPs is confined between a patterned substrate and a top plate. The top plate is then pulled relative to the templated substrate, allowing for solvent evaporation from the vapor/solvent interface at the receding edge. The flow produces a dense accumulation of NPs in the region of the receding line, which drags the accumulation zone across the substrate, and the NPs exit the meniscus to selectively fill the traps. This method can be applied to a large spectrum of materials with dimensions ranging from micrometers to nanometers and with different particle shapes, with a promise for the realization of many devices in various fields. Another colloidal assembly method, related to capillary assembly, utilizes template wrinkling to assist in the assembly of NPs.^72,73^ Here, a polymer, such as polydimethylsiloxane (PDMS), is stretched while it is oxidized in a plasma and then released to form sinusoidal wrinkles. Dropping a solution containing NPs onto the surface results in particle chains due to capillary forces that arise when the solution is dried. The particle chain patterns can be transferred to other surfaces. Other substrates, like ITO, can also be covered with a polymer, and this template is then used for the *in situ* synthesis of particles. Functional groups on the polymers can aid the confined electrochemical deposition (ECD) of NPs. The arrangement and density of the NP array is controlled by the polymer template design as well as the ECD conditions.^74^ Ag NPs have thus been patterned, and the feature sizes in the array played a role in its ability to enhance fluorescence signals. The polymer ECD method provides good positional control, as the stamping of the polymer (NIL) dictates this step. However, within each polymer patch, the NPs were found to possess a high polydispersity.^75^

Highly ordered arrays of functional molecules can also be printed using dip-pen nanolithography (DPN). One major disadvantage is that DPN is a slow serial process. With specially designed AFM-like (atomic force microscopy) tips, it is possible to “print” molecules such as bioactive proteins on to a nickel coated silicon wafer, at sizes ranging from 80 to 500 nm and at distances between 1 and 3 μm. The printing process occurs at 80% humidity, so the tip to substrate diffusion is enhanced, and the proteins conserve their activity after the patterning process.^76^ This same technique has been combined with coordination chemistry to form a stable pattern of tobacco mosaic viruses. As the surface proteins of the virus are prone to fill as many coordination sites as possible, it makes for a nearly perfect aligned pattern according to the authors of this study.^77^ Although DPN is a slow serial method, it allows for the possibility of patterning different materials, which can then be used as templates for rapid and parallel arrangement of other molecules or NPs.

An ink-free stamping method for the patterning of molecular templates is microcontact printing (μCP).^78^ For instance, a PDMS striped stamp treated with dual affinity peptide linkers can be pressed onto a substrate such as silicon oxide or gold, which transfers the peptide linkers to the surface. The peptides possess functional groups that bind to the substrate as well as functional groups that can be used to bind NPs. The PDMS stamps are prepared from a suitable large-area master that is composed of 5 μm wide stripes. Monolayers of gold NPs, silica beads, and carbon nanotubes can be deposited on the stamped patterns. Although the spacing between the stripes is controlled, the NPs themselves are not aligned or organized inside the stamped area.^79^ If e-beam patterning is used to make stamps, then the same constraints apply as in the formation of templates; for example, nanometric features are then only possible using slow e-beam writing methods and are restricted to small areas. Moreover, using a microcontact electrochemical conversion (MEC) stamp, where a localized electric field is induced by mechanically holding the modified wafer and a conductive stamp together and applying a voltage for a few minutes, the aminosilane monolayer can be oxidized at specific locations and the gold NPs are electrostatically adsorbed onto the designated patches.^80^

#### Nanopatterning Combined with Physical Vapor Deposition

Physical vapor deposition (PVD) onto a templated substrate can produce a variety of nanostructures. In particular, glancing angle deposition (GLAD), which utilizes the shadowing effect of templates by tilting the substrate during deposition, is often applied to make different nanopatterns. For example, nanometer-sized hole patterns, prepared by e-beam lithography, were used as a template substrate and resulted in unusual metal nanoring or metal nanopoint structures when combined with GLAD.^81^ The NIL technique has also been combined with GLAD, leading to large-area asymmetric nanopatterns.^82^ As mentioned in the section on nontemplated patterns, techniques like Langmuir–Blodgett and BCML can be used for the parallel fabrication of hexagonal patterns. When combined with GLAD, quasi-hexagonal patterns of particles composed of different materials, tunable sizes, and features emerge.^8,83^ Using this combination of techniques, multilayer particles can be designed and individually etched to fabricate patterns of hollow Au domes or large-area masks can be formed for NIL (Figure 5d).^84^

The development of a process where no stamp or lithography mask is required is preferable, as this can considerably scale up the use of these chemical techniques to permit the patterning of large areas and to enable industrial applications. Some examples of such efforts are introduced in the next section, where methods that use external fields for patterning are presented. A summary of the methods discussed in this section can be found in Table 2.

**Table 2:** Chemical and Physical Force Template-Assisted Nanopatterning Methods

methods	types of substrates	types of particles (materials, shape, size)	interparticle distance	reported patterned areas	refs
DNA	most solution stable substrates	NPs that can be coupled to DNA	template-dependent and in some cases solvent-dependent	template-dependent (up to 600 μm^2^)	(52−54,62,63)
wetting	most substrates	chemically addressable NPs	tens of nanometers to several micrometers	several micrometers	(47,48,85)
capillary assembly	most substrates	most particles	template-dependent	template-dependent (up to centimeter-scale area)	(68−70)
ECD	conducting substrates such as ITO	any materials that can be electrochemically deposited	template-dependent	template-dependent (up to centimeter-scale area)	(74,75)
DPN	AFM suitable, stable at high humidity; coated Si wafers	small organic molecules, proteins, particles, and viruses	several micrometers	template-dependent, several micrometers, larger area is possible	(76,77)
microcontact printing	polymer-coated chemically modified surfaces (Si wafer)	Au NPs and possibly other particles	electron lithography beam dependent	up to a square centimeter	(78−80)
PVD-assisted (chemical or physical)	most substrates	any material that can be evaporated, shape and size are dependent on template and deposition conditions	template-dependent	template-dependent (up to 3 in. wafers)	(81−84)

### Field-Assisted Patterning

Field-assisted manipulation is regarded as a powerful tool for rapidly organizing nanoscale particles into various large-area patterns. Holding and moving the smallest objects such as micro-/NPs, individual biological cells, and even atoms with magnetic or electromagnetic fields can be realized with magnetic or optical tweezers.^86−88^ Similarly, it is possible to pattern NPs into a desired array on a large-area substrate in parallel using these types of fields. Moreover, magnetic fields have assisted nanopatterning in various ways, leading to some successful patterns of micro- and NPs.^89−92^ In this section, we introduce several examples of field-assisted techniques for large-area nanopatterning.

**Figure 6:**
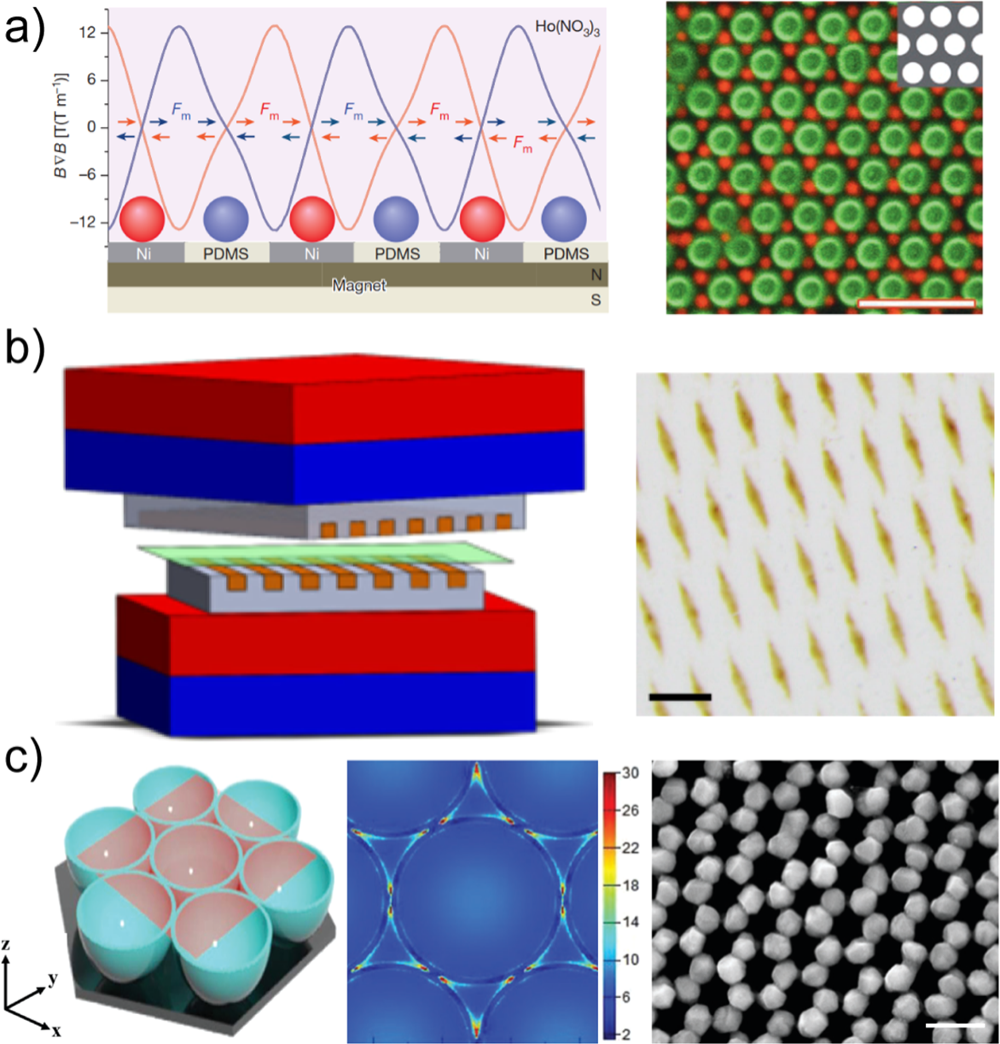
Field-assisted patterning. (a) Magnetic field-assisted nanopatterning method: (Left) Scheme of experimental arrangement and calculated force profiles. Lateral magnetic forces (arrows labeled *F*_m_) position magnetic particles (red) onto the nickel “islands” in the grid and nonmagnetic particles (blue) onto the “voids” of the grid. (Right) Paramagnetic particles (red; 1.5 μm) and nonmagnetic particles (green; 2.5 μm) on a hexagonal grid form and AB_2_ hexagonal lattice. Scale bar: 10 μm. Reproduced with permission from ref (93). Copyright 2013 Springer Nature. (b) Magnetic field-assisted interference-like patterns: (Left) Scheme of experimental setup for interfering with the fields produced by two magnetically patterned PDMS stamps placed between two permanent magnets. One of the stamp–magnet pairs is stationary, and the other is manipulated by a motorized translational and/or rotational stage. (Right) Top view of a pattern of Fe_3_O_4_ NPs formed between two arrays of lines placed at an angle of 30° with respect to one another. Particles are localized exclusively to the rhomboidal regions at the lines’ intersections. Scale bar: 200 μm. Reprinted (adapted or reprinted in part) with permission under a Creative Commons Attribution 4.0 Internationl License from ref (95). Copyright 2017 Springer Nature. (c) Light-assisted nanopatterning method: (Left) Idealized morphology of the Au nanobowls (which are the template for site-selective growth of Ag NPs) as predicted by simulation, where the light red slices represent the locations of the frequency-domain field monitor. The light source is circularly polarized with a wavelength in the range from 620 to 630 nm with a vertical incidence corresponding to the *x*–*y* plane. (Right) Scanning electron microscopy images of the Au nanobowls with Ag NPs grown at the nanobowl edges. Scale bar: 500 nm. Reproduced with permission from ref (110). Copyright 2019 RSC Publishing.

Large-area nonmagnetic and magnetic microparticles were both successfully patterned using magnetic microgradient fields in a paramagnetic fluid.^93^ The key principle is that the magnetic field, experienced by a particle, is determined by the difference in magnetic susceptibilities of the particle and of the dispersing medium as well as the particle size. By tuning these parameters and combining patterned magnetic fields on length scales commensurate with particle sizes, one can pattern both paramagnetic and diamagnetic particles. To produce the magnetic field patterns, a nickel grid with the desired periodicity was used as a template, which was embedded in a layer of PDMS and then magnetized. The Ni-PDMS composite film both concentrates and modulates the magnetic field, as shown schematically in Figure 6a. Different sizes of magnetic particles and nonmagnetic particles formed AB_2_ structures, as shown on the right of Figure 6a. The larger nonmagnetic particles are positioned on the PDMS areas, and the smaller magnetic particles only fit onto the three-fold junctions of the honeycomb grid.

Patterning by magnetic fields is rapid and precise but is often restricted to magnetic particles. However, magnetic patterning of general nanoscale materials through nonmagnetic templates has been introduced using ferrofluid-induced magnetic interactions between nonmagnetic particles and template patterns.^94^ The nonmagnetic templates, when exposed to a magnetized ferrofluid, behave as “reverse micromagnets” in an external magnetic field, owing to the difference in the magnetic properties between the templates and the surrounding ferrofluid. Then the magnetic field around the template structures is modulated to induce a large local field gradient, which attracts the nonmagnetic particles that have acquired net magnetic moments. Thus, patterning nanoscale particles on nonmagnetic templates has become possible.

Another interesting patterning method in conjunction with magnetic fields relies on interference-like patterns.^95^ Magnetic NPs contained in a polymer layer were sandwiched between two magnetic grids, as shown in Figure 6b, for the experiment. Moiré patterns were achieved due to field gradients in the plane of the polymer layer. The magnetic NPs accumulated in the “crest” regions formed by the local fields of both grids and are completely cleared from the “troughs”. By controlling the periodicity or shapes of the two templates and their orientation, various patterns can be made rapidly on large areas.

Electric fields can also be used to pattern NPs with dielectrophoresis (DEP), which utilizes a nonuniform AC electric field and polarizable particles in solution.^96,97^ For this method, lithographically prepared templates or microelectrodes are used to serve as guiding features to direct NPs on a substrate. As an example of this method, NPs that are more polarizable than the surrounding medium are placed on a template; they experience a net force toward the areas with a higher field gradient, resulting in the desired patterns.^98,99^ Also, polystyrene NPs ranging from 100 nm to 2 μm were immobilized into an array of thousands of microelectrodes at the same time, enabling a parallel patterning of NPs.^100^ The use of an electron or ion beam to form localized changes on a silicon substrate has also been demonstrated.^101^ If this substrate is thereafter immersed in a gold colloidal solution, the modifications to the chemical properties of the native silicon oxide, as well as the solution pH, and the type of beam, help fine-tune the ability to assemble the gold NPs onto the surface. The results achieved by this process range from particle distances of <100 nm to >1 μm.

Electric fields can be used to form large-area nanopatterns by a method termed electrochemical lithography (EL).^102^ Stamps formed by e-beam lithography made from conductive materials are immersed in an electrolyte and placed in close proximity to a substrate. Application of a potential leads to an electrochemical reaction on the substrate forming a pattern that is guided by the stamp. For example, nanoline patterns were formed by the oxidation of Si to SiO_2_ on an area of 1 cm^2^ in less than a minute.^103^ Furthermore, EL can be used to form thin (∼15 nm) dot, line, and mesh nanopatterns.^104,105^

Finally, light-assisted nanopatterning techniques have also been examined. Interference lithography (IL) can be used to generate regular patterns in a photoresist layer by interfering two or multiple coherent light waves. Periodic arrays of nanostructures can be patterned in combination with deposition or etching.^106−108^ IL is a parallel method that enables large-area nanopatterning, and recently, arrays of metallic sub-100 nm nanostructures have been formed.^109^ Apart from lithography methods, a light-assisted bottom-up method was also reported. For this method, gold bowl-templated substrates, which were prepared by selectively etching close-packed polystyrene beads to leave behind gold caps, were used.^110^ When these templates were covered with Ag-containing solutions, Ag NPs selectively grew on the gold sites after illumination. The illumination caused a localized surface plasmon resonance effect in the triangle between the bowls, facilitating preferential growth, as shown in Figure 6c. This work shows that light can be used to control a chemical reaction at the nanometer scale, enabling the control and design of a wide variety of nanopatterns. Another method has demonstrated the use of the Moiré effect in combination with UV illumination as a way to form patterns of quasiperiodic symmetries by rotation of simple 1D and 2D PDMS masks onto substrates covered with a photoresist material. The rotation in conjunction with multiple exposure steps at various angles produces arrays with rotational symmetries that are higher than the ones present in the PDMS mask. The achievable pattern dimensions, up to several inches, are limited by the size of the mask and not by the Moiré nanolithography.^111^

A technique combining the use of an electrical field to manipulate particles and a light field to fix particles was recently introduced.^112^ Electric manipulation demonstrates a high degree of control and precision, and it can be applied to NPs made of any materials including metals, semiconductors, and insulators. Here, both uniform DC and AC electric fields were used. The DC field moves particles by Coulomb interactions, and the AC field exerts a torque for angular orientation. Using patterned quadrupole microelectrodes combining DC and AC electric fields, particles can be transported to a designated position and aligned. Once a particle is positioned, a light-triggered click reaction is used to fix it on the substrate. For example, sulfur-rich MoS_2_ nanoribbons react with molecular thiol groups under UV exposure, which allows the particles to be fixed on the substrate by UV click chemistry.^113^ In this way, particles can be positioned on the substrate in desired patterns without templates. A summary of all field-assisted patterning techniques can be seen in Table 3.

**Table 3:** Field-Assisted Nanopatterning Methods

methods		types of substrates	types of particles (materials, shape, size)	interparticle distance	reported patterned areas	refs
magnetic field	direct assembly	nickel grid as a template and PDMS as medium	superpara- and diamagnetic particles (nonmagnetic particles possible), spheres, 800 nm to 3.2 μm	several micrometers (template-dependent)	1 cm^2^	(93)
	ferrofluid-assisted	polyurethane pattern as a template and magnetized ferrofluids as a medium	nonmagnetic particles, spheres, microbeads (1–10 μm)	several tens of μm (template-dependent)	template-dependent	(94)
	interference	PDMS-containing magnetic/nonmagnetic particles as stamps and PMMA film as the medium	magnetic particles (Fe_2_O_3_), spheres, NPs (12 nm)	clusters of particles, distance between clusters is 100–200 μm	∼25 mm^2^ (template-dependent)	(95)
electric-field-assisted	DEP	array of microelectrodes embedded in a SiO_2_ matrix	most materials, spheres, 100 nm to 2 μm	∼2 μm (template-dependent)	∼100 μm (template-dependent)	(100)
	EL	either conductive or substrates that can react electrochemically	materials formed by electrochemical reactions (*e.g.*, oxides), ∼15 nm to several micrometers	stamp or microcahnnel dependent	∼1 cm^2^	(102−105)
light	interference	Au film deposited glass	photoresist (Au film is etched based on the photoresist pattern)	template-dependent	4 cm^2^	(109)
	light-assisted chemistry	Au nanobowl (plasmonic materials) as a template and a precursor containing solution	Ag particles, (precursor-dependent), sphere-like shape, several tens of nanometers (time-dependent)	500 nm of hexagon rings (template-dependent)	1 cm^2^ (template-dependent)	(110)
combining electric field and light		glass substrates with thiol groups	most materials, functionalized surface for UV click chemistry	precisely controlled by electric-field (not limited)	500 μm^2^	(112)

## Conclusions and Outlook

Directing the assembly of NPs to form regular patterns on surfaces is not an easily achieved task. The use of parallel patterning methods is essential if large areas are to be patterned. The quality of the pattern and the interparticle spacing are important parameters for a number of applications, such as plasmonics, optics, and electronics. A formidable challenge is the ability to control the NP assembly with precision and form patterns reproducibly. The challenges are due to low efficiency or the inevitable structural defects. The combination of self-assembly strategies in conjunction with existing nanofabrication techniques could potentially provide effective solutions for fabricating larger nanopatterns more controllably.

Close-packed NP arrays are fundamentally simpler to fabricate, as most of the fabrication methods rely on self-assembly of the particles that form the pattern, which also alleviates the need for templates. For close-packed patterns, there is no need to consider the distances between the particles; as such, no extra modification of the particles is necessary. While this form of patterning is well advanced, there is a need to allow the patterning of complex-shaped (nonspherical) nanostructures and to permit the distances to be tuned.

More control, especially over the interparticle separation, is afforded by more complex fabrication techniques. In most cases, these are based on a prefabrication step, such as the writing/preparation of a template structure. Lithography methods for the development of templates are commonly used. When submicron accuracies are needed, they represent a hindrance, as they involve time-consuming slow processes, such as electron-beam lithography, dip-pen nanolithography, or thermal scanning probe lithography, but the precision achieved often justifies their use. If the template can be used multiple times, as in stamping or nanoimprint lithography, then larger-scale applications can also be realized by these methods.

Particularly promising for larger-scale applications are methods that rely on self-assembly or that are based on chemical interactions, magnetic or electrical fields, and interactions with light to form a template. However, due to the complexity of these methods, the main focus of the applications is still in the laboratory environment and used mainly for research purposes. Further industrial applications require the development of advanced processes, where no stamps or lithography masks are required, which would increase the use of chemical techniques for even larger-scale patterns and indeed advance the use of these nanopatterns for industrial applications.

Future developments are likely to advance hierarchically stacked 2D nanopatterns to form 3D structures. IL is already being used to realize 3D photonic crystals with the help of multibeam interference.^114,115^ However, bottom-up methods, for instance, using acoustic fields, also promise a route to form 3D structures *via* “one-shot” assembly.^116−118^ Advanced PVD techniques, such as GLAD, can also be applied to obtain hierarchically stacked patterns on large areas.^8^

Development of transfer techniques is also important for future applications to overcome the limitations of possible patterning materials and substrates. For example, the nanotransfer printing (nTP) technique was introduced to transfer nanopatterns to highly curved surfaces, which is not possible by conventional lithography methods.^119^ After the first report, various innovative nTP techniques have been reported including transfer to nonplanar and flexible substrates.^120,121^ Very recently, transferred patterns with sub-20 nm resolution and 8 in. wafer scalability were achieved.^122^ Further development will enable some special functionalities of materials or devices by removing the constraints of patterning materials and substrates.

In addition to the currently reported methods, there can be a myriad of other creative methods. There is a great possibility that more complex and desired patterns can be formed on a large area with excellent material and substrate compatibility. Through a smart design of patterning methods, both structural and functional complexity could be achieved. Therefore, the collaboration between researchers from different fields would be of great value to achieve on-demand large area patterns.

## Vocabulary

**nanopattern**: a pattern of nanostructures or nanoparticles organized in a periodic manner on a solid substrate
**serial method/parallel method**: a fabrication method is described as serial when features are made one after the other in sequence, while a parallel fabrication method forms multiple or even all features simultaneously
**close/non-close-packed**: in a close-packed pattern, the features are in contact with each other, and for the non-close-packed, there is space between them
**templated substrate**: a substrate that is treated before the actual patterning process, on top of which a stencil is formed, which is used to guide the arrangement of the particles
**chemical assisted**: the arrangement of the nanostructures in to the nanopatterns is controlled by a molecular interactions or chemical reactions
**field assisted**: the arrangement of the nanostructures in to nanopatterns is controlled by electromagnetic fields
